# Individual differences in resting heart rate variability and cognitive control in posttraumatic stress disorder

**DOI:** 10.3389/fpsyg.2014.00758

**Published:** 2014-07-15

**Authors:** Brandon L. Gillie, Julian F. Thayer

**Affiliations:** Department of Psychology, Ohio State UniversityColumbus, OH, USA

**Keywords:** cognitive control, individual differences, heart rate variability, posttraumatic stress disorder

## Abstract

Post-traumatic stress disorder (PTSD) is characterized by deficits in cognitive functioning, particularly cognitive control. Moreover, these deficits are thought to play a critical role in the etiology and maintenance of core PTSD symptoms such as intrusive thoughts and memories. However, the psychophysiological concomitants of cognitive control remain largely unexamined. In this article, we suggest that individual differences in heart rate variability (HRV), a physiological index of self-regulatory capacity, may underlie the association between cognitive control ability and intrusive cognitions in PTSD. We review evidence showing that individual differences in HRV at rest are related to prefrontal cortical activity and performance on a broad range of cognitive control tasks. We highlight the importance of inhibition as a mechanism by which HRV promotes successful cognitive control. In addition, we summarize recent research linking individual differences in HRV to performance on laboratory tasks that assess the ability to control unwanted memories and intrusive thoughts. We conclude by suggesting that future studies should examine the role of low HRV as a risk factor for developing PTSD.

## INTRODUCTION

Traumatic experiences can greatly alter an individual’s cognitive, emotional, and physiological functioning as demonstrated by those with post-traumatic stress disorder (PTSD), who experience avoidance, hyperarousal, and re-experiencing symptoms (American Psychiatric Association, 2013). While current models of PTSD have emphasized the role of psychological processes such as attentional bias toward potential threat, enhanced recall of trauma-relevant material, and appraisal of the trauma event and its sequelae as mechanisms that underlie PTSD symptom expression (for review, see Ehlers and Clark, 2000; Brewin and Holmes, 2003), there has been less focus on the role of cognitive control. However, researchers have recently suggested that the re-experiencing symptoms of PTSD, such as intrusive thoughts and memories, may stem from deficits in cognitive control that exist prior to the onset of trauma (Dalgleish et al., 2008; Levy and Anderson, 2008; Bomyea et al., 2012a). Yet, the physiological underpinnings of such a relationship remain unclear. We propose that individual differences in heart rate variability (HRV), a physiological index of self-regulatory capacity, may underlie the association between cognitive control ability and intrusive cognitions in PTSD. To begin, we briefly summarize the model of neurovisceral integration (Thayer and Lane, 2000, 2009), which suggests a role for individual differences in HRV in the regulation of cognitive processes.

## HEART RATE VARIABILITY: THE NEUROVISCERAL INTEGRATION MODEL

Thayer and Lane (2000, 2009) developed the neurovisceral integration model, which suggests that individual differences in vagal function, as indexed by heart rate variability (HRV) at rest, reflect the activity of a flexible and integrative neural network, which allows the organism to effectively organize emotional, cognitive, and behavioral responses in the service of goal-directed behavior and adaptation. An important part of the neurovisceral integration model is the complex interplay between cortical and subcortical regions collectively termed the central autonomic network (CAN; Benarroch, 1993). The CAN serves as the neuroanatomical link between the autonomic nervous system and brain areas associated with higher order cognitive functioning (e.g., the prefrontal cortex). The neural structures of the CAN include the anterior cingulate, the insula, the ventromedial prefrontal cortices, the central nucleus of the amygdala, the paraventricular and related nuclei of the hypothalamus, the periaquaductual gray matter, the nucleus of the solitary tract (NTS), the nucleus ambiguous, and the medullary tegmental field, among others (Thayer and Lane, 2009; Thayer et al., 2012). These components are reciprocally interconnected, allowing the prefrontal cortex to exert inhibitory control over subcortical structures in order to generate cognitive, behavioral, and physiological responses that support goal-directed behavior and adaptability. Critically, the output of this inhibitory cortico-subcortical circuit extends to autonomic inputs to the heart, including the vagus nerve. In this model, higher levels of HRV (i.e., greater vagal tone) at rest are a product of a system in which the prefrontal cortex exerts inhibitory control over subcortical circuits thus allowing the organism to respond to environmental challenges in a controlled and adaptive manner when needed. For this reason, examining the parasympathetic influence on the heart via HRV can provide an index of an individual’s capacity to effectively function in a complex and challenging environment.

Studies using pharmacological and neuroimaging approaches demonstrate that prefrontal cortical activity is associated with vagally mediated HRV (Ahern et al., 2001; Lane et al., 2009; Thayer et al., 2012). For example, pharmacological inactivation of the prefrontal cortex increased heart rate and decreased vagally mediated HRV (Ahern et al., 2001). These findings establish that the prefrontal cortex tonically inhibits cardioacceleratory circuits and changes in cortical activity are reflected in HRV. Neuroimaging studies have linked HRV to activity in a number of prefrontal brain regions including the ventromedial prefrontal cortex, superior prefrontal cortex, and dorsolateral prefrontal cortex (Lane et al., 2009); these associations were further supported by a recent meta-analysis of neuroimaging studies that included HRV (Thayer et al., 2012). Together, these findings provide a conceptual model of individual differences in HRV as a marker of self-regulatory capacity. Next, we review evidence linking HRV at rest to specific cognitive control functions including attention, working memory, and inhibition.

## INDIVIDUAL DIFFERENCES IN HRV AND COGNITIVE CONTROL ABILITY

Cognitive control refers to the mental processes involved in keeping desired information active while inhibiting irrelevant or unneeded information (Braver, 2012). While cognitive control is subsumed within the broader construct of “executive function,” the general purpose control mechanisms that regulate thoughts and behaviors (Miyake and Friedman, 2012), it may not reflect the capacity for higher order functions, such as organization, sequencing, reasoning. Miller and Cohen (2001) proposed that successful cognitive control stems from the active maintenance of patterns of activity in the prefrontal cortex that represent goals and the means to achieve them. In addition, the extent to which prefrontal control areas exhibit greater connectivity with other functional networks is associated with increased cognitive control ability. Given that resting HRV has been shown to index important aspects of prefrontal neural function, it follows that individual differences in HRV may be a useful predictor of cognitive control ability. It is worth noting that cognitive control is not a unitary construct. In support of this idea, Miyake et al. (2000) used latent-variable analysis to identify three specific functions that underlie the construct of cognitive control: information *updating and monitoring, attentional set-shifting*, and *inhibition*. However, recent perspectives suggest that inhibition may be a “basic function” that underlies other aspects of cognitive control (Thayer et al., 2009; Miyake and Friedman, 2012). Put another way, control over working memory and attentional set-shifting may require some degree of inhibition. Conceptual views of HRV parallel these ideas, as inhibition is seen as the core mechanism by which individuals produce context appropriate responses; individual differences in HRV reflect the extent to which these inhibitory processes are effective (Thayer, 2006). Thus, although HRV is largely an index of inhibitory control, it should also be associated with performance-based measures of working memory and attention.

A growing body of research has found that individuals with higher levels of HRV at rest demonstrate enhanced performance on cognitive control tasks that require working memory, attentional control, and inhibition. Hansen et al. (2003) found that individuals with higher levels of HRV performed better on a standard two-back working memory task compared to those with lower levels of HRV. Another study replicated and extended these results by showing that high HRV individuals maintained enhanced working memory capacity even in the context of a stressful environment (i.e., under threat of shock; Hansen et al., 2009). Moreover, aerobic training/detraining produced concomitant changes in working memory performance and HRV; those who continued aerobic training over a 4-week period showed increased accuracy on a working memory task and higher levels of HRV post-task relative to those who discontinued exercise (Hansen et al., 2004). These findings provide support for a causal relationship between individual differences in HRV and working memory capacity.

Resting levels of HRV are also associated with performance on tasks that require attentional control (Park and Thayer, 2014). Park et al. (2012) found that individuals with low levels of HRV were less able to inhibit their attention away from locations where fearful faces were previously presented. A subsequent study demonstrated that the previous findings likely reflected both automatic and voluntary deficits in attentional control as those with low HRV showed increased attentional engagement to and decreased disengagement from fearful faces (Park et al., 2013). In addition, among individuals with dental anxiety, those with lower levels of HRV were less able to regulate their attention when presented with threat-related dental words relative to those with higher levels of HRV (Johnsen et al., 2003). Thus, individual differences in HRV are associated with the capacity to control attention, especially in the presence of emotional stimuli.

Consistent with perspectives that suggest a relationship between prefrontal inhibitory processes and HRV (Thayer and Lane, 2000, 2009), studies have found associations between individual differences in HRV and performance on tasks that require motor response-inhibition and inhibitory control more broadly. Using an emotional stop-signal task that required individuals to withhold their motor response in the presence of negative emotional cues, those with higher levels of HRV activated and inhibited their responses faster than those with lower levels of HRV (Krypotos et al., 2011). A recent study by Hovland et al. (2012) found that higher levels of HRV at rest were associated with better performance on the *Wisconsin Card Sorting Task* and the *Color-Word Interference Task*, two measures of general cognitive flexibility and executive function. Importantly, although resting HRV predicted general performance on the tasks, it was most strongly associated with aspects of the tasks that reflected inhibitory control (Hovland et al., 2012). Altogether, there is considerable evidence suggesting that individual differences in HRV are linked to the specific mental processes that underlie cognitive control, particularly inhibition.

## COGNITIVE CONTROL DEFICITS IN PTSD

Deficits in cognitive control that have been observed in patients with PTSD appear to parallel those that have been associated with individual differences in HRV. However, it is worth noting that the association between cognitive control ability and PTSD is complex and evidence for broad cognitive control deficits in PTSD remains mixed. For example, while some have found that those with PTSD perform worse on neuropsychological measures that require a combination of sustained attention, inhibition of habitual responses, and set shifting, compared to trauma-exposed individuals without PTSD and healthy controls (Stein et al., 2002; Polak et al., 2012; Cohen et al., 2013) others have failed to show such associations (Barrett et al., 1996; Crowell et al., 2002; Kanagaratnam and Asbjørnsen, 2007). Moreover, some have noted that comorbid conditions such as depression, anxiety, and substance abuse, rather than PTSD symptoms, may account for group differences in cognitive control ability between those with PTSD and healthy controls (Barrett et al., 1996). In light of these findings, researchers have begun to investigate whether PTSD may be best characterized by deficits in specific cognitive control functions, rather than widespread cognitive impairment (Leskin and White, 2007; Aupperle et al., 2012).

Relative to other aspects of cognitive control, deficits in inhibitory control have been most consistently observed among individuals with PTSD (Aupperle et al., 2012). For example, Leskin and White (2007) found that while college undergraduates with PTSD were less able to inhibit their attention to irrelevant distractors relative to trauma-exposed controls, the groups performed similarly on tasks assessing attentional set-shifting, alerting, and orienting. Similarly, others have shown that those with PTSD perform significantly worse than healthy controls on tasks that require inhibitory control (e.g., the stroop test) but not those tasks that assess attention span and working memory capacity (Flaks et al., 2014). Moreover, individuals with PTSD show deficient motor response control as evidenced by high inhibition-related error rates on the Go/No-Go and Stop-Signal tasks (Casada and Roache, 2005; Falconer et al., 2008; Wu et al., 2010; Swick et al., 2012). Because inhibition is often required for adaptive self-regulation, individual differences in inhibitory control should be associated with PTSD severity.

Indeed, evidence suggests that deficits in inhibitory control are associated with elevated PTSD symptoms, particularly the experience of unwanted memories and thoughts (Vasterling et al., 1998; Leskin and White, 2007; Bomyea et al., 2012a). Laboratory investigations have focused on the relationship between proactive interference control, an inhibition-related function that taps the ability to resist information that was previously relevant to the task but has since become irrelevant (Friedman and Miyake, 2004), and the frequency of intrusive memories and thoughts. Those who display lower levels of proactive interference control, assessed through a variety of neuropsychological tests, self-report a greater frequency of intrusive thoughts (Friedman and Miyake, 2004; Bomyea et al., 2012a). Individual differences in proactive interference control also play a role in the experience of unwanted thoughts and memories following stressful events. Wessel et al. (2008) found that greater proactive interference control predicted less self-reported intrusive cognition 24 h after viewing an emotionally evocative trauma film clip. Others have shown that the relationship between proactive interference control and intrusion frequency after stress is consistent across longer time intervals (1 week) and cannot be accounted for by neuroticism and gender (Verwoerd et al., 2011). These findings provide initial evidence that deficits in inhibitory control may serve as a vulnerability factor for experiencing intrusive thoughts and memories. A related topic is the extent to which individual differences in cognitive control correlate with the effectiveness of attempts to control intrusions via mental control strategies.

Along these lines, researchers have investigated the association between cognitive control ability and suppression, a strategy commonly used to manage the experience of intrusive cognitions. Suppression is widely considered to be a maladaptive strategy as it sometimes serves to paradoxically increase the frequency of unwanted cognitions (for review see Wenzlaff and Wegner, 2000). Attempts at thought suppression among those with PTSD are often unsuccessful as evidenced by a paradoxical increase in trauma-related intrusive thoughts following instructed suppression in a laboratory setting (Shipherd and Beck, 1999, 2005). In a similar manner, individuals with PTSD who attempt to suppress trauma memories typically experience enhanced remembering of the trauma and other negative personal material, as well as a lack of specificity in the recollection of the personal past (Dalgleish et al., 2008). Ineffective suppression of unwanted thoughts and memories is associated with deficient cognitive control ability. For example, individuals with reduced working memory capacity are less able to suppress intrusive thoughts relative to those with higher levels of working memory capacity (Brewin and Beaton, 2002; Brewin and Smart, 2005). In addition, those with low levels of inhibitory control show a reduced capacity to stop retrieval of unwanted memories (Depue et al., 2010; Wessel et al., 2010). Dalgleish et al. (2007) have shown that reduced specificity of autobiographical memory, a consequence of unsuccessful memory suppression, is largely a function of reduced cognitive control. Thus, individual differences in cognitive control may influence both the tendency to experience intrusive cognitions and the extent to which such intrusions are controllable.

While there is growing evidence suggesting that the re-experiencing of symptoms and intrusive cognitions experienced by individuals with PTSD are associated with deficits in cognitive control, two issues remain unsettled. One question concerns the nature and organization of cognitive control deficits observed in PTSD. Specifically, it remains unclear whether these deficits are the result of disruptions in broadband cognitive ability or impairment in more specific functions such as inhibition. Another issue is that few studies have examined the psychophysiological correlates of cognitive control ability among those with PTSD. Adopting a psychophysiological perspective may provide greater insight into the relationship between cognitive control and the severity of PTSD symptoms and lead to new research directions focused on the etiology and treatment of PTSD. Individual differences in resting levels of HRV are associated with cognitive control ability, particularly inhibitory processes. Moreover, the neurovisceral integration model suggests that low HRV at rest may serve as an endophenotype for some forms of psychopathology, including anxiety disorders (Melzig et al., 2009; Thayer and Lane, 2009). By examining individual differences in resting HRV, researchers may be better able to elucidate the relationship between cognitive control ability and re-experiencing symptoms among those with PTSD. One interesting possibility is that individual differences in HRV may underlie the association between cognitive control ability and intrusive cognitions in PTSD. Although this idea has yet to be directly examined, researchers have begun to recognize the role of autonomic dysfunction (i.e., low HRV) among those with PTSD. In addition, studies have started to investigate associations among individual differences in HRV, cognitive control processes, and re-experiencing symptoms. Next, we review evidence demonstrating that individuals with PTSD tend to be characterized by low resting HRV. In addition, we show that low resting HRV is also associated with deficits in cognitive control processes and the tendency to re-experience unwanted thoughts and memories.

## ASSOCIATIONS AMONG HRV, PTSD, AND RE-EXPERIENCING SYMPTOMS

Given that individual differences in HRV index the degree to which the prefrontal cortex exerts a tonic inhibitory influence over subcortical circuits, one would expect that disorders characterized by psychological inflexibility and impaired inhibitory control, as is the case for PTSD, would also be associated with low resting levels of HRV. Indeed, a number of studies show that individuals with PTSD display lower levels of HRV at rest compared to healthy controls and trauma-exposed individuals without PTSD (Cohen et al., 1998; Sack et al., 2004; Jovanovic et al., 2009; Hauschildt et al., 2011; Nagpal et al., 2013; Norte et al., 2013). Blechert et al. (2007) found that individuals with PTSD exhibited lower levels of vagally mediated HRV at rest relative to healthy controls and patients with panic disorder, another anxiety disorder also characterized by low resting HRV (Friedman and Thayer, 1998). Importantly, other investigations have shown that individuals with PTSD are characterized by reduced levels of HRV even after accounting for important covariates such as traumatic brain injury and levels of depression (Minassian et al., 2014). A limitation of the extant literature is that a majority of studies are cross-sectional. Thus, it is unclear whether reduced levels of HRV observed in patients with PTSD represent a pre-trauma vulnerability factor or result from exposure to trauma. However, given that resting levels of HRV appear to be relatively stable over time (Li et al., 2009), it seems more likely that low resting levels of HRV may precede the onset of a traumatic event. In support of this idea, evidence suggests that low levels of HRV prospectively predict increases in anxiety among women diagnosed with breast cancer (Kogan et al., 2012). In addition, a recent study found that HRV measured soon after trauma exposure predicted the development of PTSD 6 months later; those with lower vagally mediated HRV at rest were more likely to develop PTSD and show greater severity of symptoms relative to those with higher vagally mediated HRV (Shaikh al arab et al., 2012).These findings provide initial support for the notion that low HRV at rest increases an individual’s vulnerability to develop PTSD. If having low HRV increases an individual’s susceptibility to develop PTSD, it may do so by way of its relationship to cognitive control processes.

Individual differences in HRV influence the effectiveness of cognitive control processes involved in managing the experience of intrusive memories and thoughts. A recent study (Gillie et al., 2014) examined whether HRV at rest predicted the degree to which individuals are able to suppress unwanted memories, assessed via the Think/No-Think Task (Anderson and Green, 2001). In this task, participants learn a series of a word pairs and later intentionally and repeatedly attempt to stop retrieval of the memory of the words when presented with a cue. Successful suppression of a target memory should reduce its accessibility at a later point; therefore, recall for the response words is assessed at the end of the experiment. Moreover, effective suppression is thought to require a high degree of inhibitory control (Levy and Anderson, 2008). Gillie et al. (2014) found that higher levels of resting HRV were associated with more successful suppression, as indicated by lower recall of the to-be-suppressed stimuli relative to control stimuli. Another study by this group examined the association between HRV and control over unwanted thoughts using a standard laboratory thought suppression paradigm (Gillie et al., submitted for publication). Participants were randomly assigned to either a suppression or free-thought control condition and asked to monitor the occurrence of a personally relevant intrusive thought. Among those instructed to suppress, higher levels of HRV were associated with greater declines in thought intrusions across the monitoring periods. Moreover, when HRV was low, higher spontaneous suppression effort ironically predicted greater persistence of intrusive thoughts over time. Taken together, these findings demonstrate an association between individual differences in HRV and the ability to exert control over unwanted thoughts and memories. It is worth noting that these studies included only healthy, college-aged participants. Thus, it remains to be seen whether these findings generalize to individuals with PTSD.

## CONCLUSION AND FUTURE DIRECTIONS

A large body of evidence suggests that PTSD is characterized by cognitive control deficits, which in turn have been linked to the re-experiencing of symptoms such as intrusive thoughts and memories. Moreover, these deficits in cognitive control are primarily the result of impaired inhibitory processes. Building from the common neural basis for cognitive regulation and physiological regulation of the autonomic nervous system, the neurovisceral integration model suggests that individual differences in HRV may be a peripheral marker of cognitive control ability. Indeed, low resting HRV is associated with poorer performance on tasks that require cognitive control processes, especially those that tap inhibition. Initial findings suggest that individual differences in HRV may also index the extent to which individuals are subject to re-experiencing symptoms such as intrusive thoughts and memories. Understanding the relationships among individual differences in HRV, cognitive control, and re-experiencing symptoms is critical, as it may help to refine theoretical models describing the etiology and maintenance of PTSD. Perhaps more importantly, identifying and manipulating the mechanisms that underlie PTSD symptomology may lead to improvements in preventative and therapeutic approaches. Because HRV is able to index the activity of brain networks that support goal-directed behavior, some have advocated its use as a research tool to better understand basic cognitive and psychopathological processes, such as those involved in the etiology and maintenance of PTSD (Appelhans and Luecken, 2006; Thayer and Lane, 2009). We echo this statement and posit that individual differences in HRV play a central role in the relationship between cognitive control and re-experiencing symptoms among those with PTSD. The evidence reviewed in this article suggests interesting possibilities for future research.

Identifying risk factors for developing PTSD has been a major focus of previous research. A number of studies have found that particular biological and cognitive individual characteristics observed prior to the onset of trauma heighten an individual’s risk of developing PTSD (for review, see Bomyea et al., 2012b). Among the pre-trauma cognitive vulnerabilities, proactive interference, a type of inhibitory process, has been specifically linked to the occurrence of re-experiencing symptoms (Verwoerd et al., 2011). Given that HRV taps an individual’s capacity for effective inhibitory processing, it seems plausible that reduced HRV may promote poor proactive interference control which could in turn lead to more severe PTSD symptomology, especially intrusive thoughts and memories. As mentioned previously, a recent study found that HRV measured soon after trauma exposure predicted PTSD development and severity (Shaikh al arab et al., 2012). Future studies should aim to replicate and extend these findings by prospectively investigating the relationship between individual differences in HRV and cognitive control ability both before and after trauma exposure. By using prospective study designs, researchers can more fully investigate the causal pathway between HRV and the severity of PTSD symptom expression and perhaps better understand the underlying mechanisms (e.g., poor cognitive control).

The associations among HRV, cognitive control, and re-experiencing symptoms may also help to inform treatments for PTSD. If low HRV acts as a vulnerability factor for developing PTSD in the manner that we suggest, it follows that enhancing HRV through medical or psychological interventions may promote more effective cognitive control and thus reduced occurrence of re-experiencing symptoms. A number of interventions including mindfulness mediation (Tang et al., 2009), applied biofeedback (Tan et al., 2011), and aerobic exercise (Jurca et al., 2004) have been shown to increase HRV over either short-term or longer term intervals. Of particular interest are studies demonstrating that improvements in HRV as a result of aerobic fitness training are associated with concomitant increases in cognitive control ability (Luque-Casado et al., 2013; Alderman and Olson, 2014). In addition, Hansen et al. (2004) found that aerobic detraining decreased both levels of HRV and cognitive performance. These findings provide further support for the association between HRV and cognitive control and demonstrate that exercise training may influence both factors. Yet, few studies have examined whether inventions attempting to improve HRV and cognitive control affect PTSD symptom severity (but see Tan et al., 2011). Thus, future studies should examine whether interventions designed to increase resting HRV also enhance cognitive control ability and reduce PTSD symptom severity.

In this review, we sought to highlight the associations among individual differences in HRV, cognitive control, and re-experiencing symptoms that characterize PTSD. Empirical evidence suggests that these factors are intimately related, though we emphasize the importance of considering HRV given its role as a peripheral marker of organism adaptability and self-regulatory capacity. We hope that our suggestions, derived from a model of neurovisceral integration, will help researchers to develop hypotheses regarding the etiology and maintenance of PTSD symptoms.

## Conflict of Interest Statement

The authors declare that the research was conducted in the absence of any commercial or financial relationships that could be construed as a potential conflict of interest.
